# Factors Associated with Hospital Length of Stay After Coronary Artery Bypass Grafting: Perioperative Characteristics and Postoperative Complication Burden

**DOI:** 10.3390/healthcare14182968

**Published:** 2026-09-11

**Authors:** Safa Özçelik, Ünsal Vural

**Affiliations:** Department of Cardiovascular Surgery, Dr. Siyami Ersek Thoracic and Cardiovascular Surgery Training and Research Hospital, Istanbul 34668, Türkiye; unsalvural@gmail.com

**Keywords:** coronary artery bypass grafting, length of stay, postoperative complications, postoperative atrial fibrillation, EuroSCORE II

## Abstract

**Background:** We evaluated factors associated with postoperative hospital length of stay (LOS) after elective isolated coronary artery bypass grafting (CABG), including contemporaneous associations with postoperative complications. **Methods:** This single-center retrospective cohort included 1493 patients undergoing elective isolated CABG from January 2020 to September 2025. Among 1449 30-day survivors, cumulative postoperative hospital days were modeled using negative binomial regression; LOS > 7 days was the data-derived, center-specific binary outcome. Secondary models assessed postoperative events. Exploratory mortality analysis used Firth logistic regression with bootstrap validation. **Results:** Median postoperative LOS was 7 days; 406 survivors (28.0%) had LOS > 7 days. Longer LOS was associated with higher body mass index (IRR per 5 kg/m^2^, 1.05), diabetes (IRR, 1.07), hypertension (IRR, 1.09), chronic obstructive pulmonary disease (IRR, 1.21), lower left ventricular ejection fraction (IRR per 5-percentage-point decrease, 1.04), lower hemoglobin (IRR per 1 g/dL decrease, 1.04), and longer cardiopulmonary bypass (CPB) duration (IRR per 30 min, 1.11). Infection (IRR, 1.46), postoperative atrial fibrillation (IRR, 1.19), and prolonged mechanical ventilation (IRR, 1.54) were contemporaneous correlates. In exploratory mortality analysis, EuroSCORE II (odds ratio per doubling, 2.01) and CPB duration (odds ratio per 30 min, 1.78) were associated with mortality; the optimism-corrected area under the curve was 0.799. EuroSCORE II alone showed moderate discrimination. **Conclusions:** LOS reflected baseline reserve, operative burden, and postoperative complications. Without event timing, associations with postoperative events are hospitalization-level and neither prospective nor causal.

## 1. Introduction

Hospital length of stay (LOS) following coronary artery bypass grafting (CABG) is an important indicator not only of clinical recovery but also of hospital resource utilization and surgical service capacity. Prolonged hospitalization may constrain surgical throughput, particularly in high-volume centers, by increasing bed occupancy and staffing requirements. Large database studies have demonstrated substantial interhospital variation in LOS even after adjustment for patient characteristics [1]. Although preoperative variables explain only part of this variation, postoperative events provide additional information regarding the clinical course and burden of care [2]. Therefore, separate yet complementary evaluation of preoperative and operative characteristics associated with LOS and the burden of postoperative care is important for local resource planning and the development of integrated enhanced recovery pathways [3].

Nevertheless, the evidence regarding factors associated with LOS after CABG remains heterogeneous. A recent systematic review identified 56 candidate factors across 20 studies [4]. Previous studies have varied in sample size, clinical setting, outcome definition, and whether they evaluated intensive care unit or total hospital stay [5,6,7,8,9]. Moreover, LOS has frequently been dichotomized. Although threshold-based classification is straightforward to interpret, it may result in loss of information and may separate clinically similar patients around an arbitrary cutoff [10]. Direct modeling of hospital days, with threshold-based analysis used as a complementary approach, enables a more comprehensive assessment of the burden of hospitalization.

Postoperative atrial fibrillation (POAF), infection, and prolonged mechanical ventilation are common events associated with increased care requirements after cardiac surgery. POAF occurs following approximately 25% of cardiac surgical procedures [11] and has been associated with various patient- and procedure-related characteristics [12,13,14,15]. These events reflect the clinical burden during recovery. Evaluating them separately allows preoperative and operative characteristics associated with LOS to be distinguished from contemporaneous postoperative correlates.

The primary aim of this study was to evaluate the adjusted associations of prespecified preoperative and operative factors with cumulative postoperative hospital days among patients who survived to 30 days. Cumulative hospital days were modeled as the primary count outcome, whereas LOS > 7 days was evaluated in the same survivor cohort as a center-specific complementary binary outcome for prolonged hospitalization. Secondary analyses described the hospitalization-level contemporaneous associations of POAF, infection, and prolonged mechanical ventilation with LOS and also evaluated factors associated with POAF, infection, and 30-day mortality. EuroSCORE II was examined solely as an auxiliary local measure of preoperative mortality risk and was not included in the LOS models.

## 2. Materials and Methods

### 2.1. Study Design and Population

**Figure 1:**
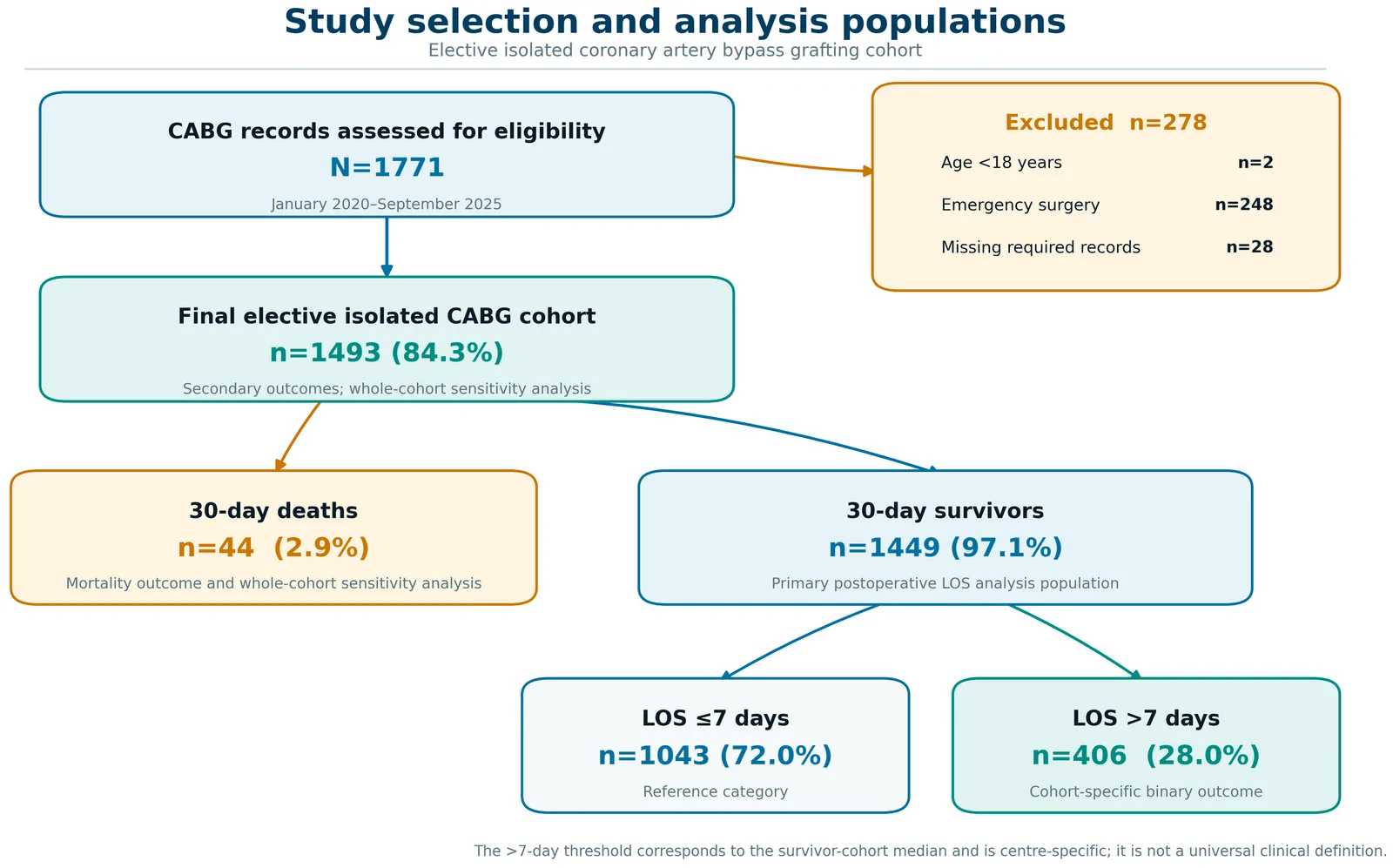
Study selection and analysis populations.

This single-center retrospective cohort included patients aged ≥ 18 years who underwent elective isolated CABG at Dr. Siyami Ersek Thoracic and Cardiovascular Surgery Training and Research Hospital between January 2020 and September 2025. Of the 1771 patients assessed, 278 were excluded: 2 were younger than 18 years, 248 underwent emergency surgery, and 28 had missing essential records. The final cohort comprised 1493 patients (Figure 1). Because the excluded records did not contain standardized, analysis-ready covariate data, a formal comparison between included and excluded patients could not be performed. No a priori sample size calculation was performed because all patients who met the eligibility criteria during the study period were included. The study was reported in accordance with the STROBE recommendations.

All procedures were performed via median sternotomy using cardiopulmonary bypass (CPB) and cardioplegic arrest. Decisions regarding graft selection, myocardial protection, perioperative support, extubation, transfer to the intensive care unit, and hospital discharge were made by the responsible clinical teams in accordance with routine institutional practice.

### 2.2. Data, Variables, and Outcomes

Electronic medical records and operative, anesthesia, perfusion, intensive care unit (ICU), laboratory, echocardiographic, discharge, and 30-day follow-up records were reviewed. Deidentified data were checked for units, coding, logical consistency, and clinical plausibility; body mass index (BMI) and creatinine clearance (CrCl) were recalculated. There were no missing data across the 31 source variables for the 1493 patients, and neither imputation nor winsorization was performed. The number of available observations for each source and derived variable is presented in Supplementary Table S7. Source records were re-reviewed to classify the anatomical pattern of every documented postoperative infection, verify the year of surgery, and retrieve four variables: documented smoking status, number of grafts, recorded number of red blood cell (RBC) units transfused, and cerebrovascular events. These fields were complete for all 1493 patients. Patient-level reconciliation confirmed the consistency of the 25 prespecified analysis variables across data extractions.

Baseline variables included age, sex, BMI, diabetes mellitus, hypertension, chronic obstructive pulmonary disease (COPD), left ventricular ejection fraction (LVEF), serum creatinine, hemoglobin (Hb), year of surgery, documented smoking status, and EuroSCORE II. CrCl was calculated using the Cockcroft–Gault equation, and chronic kidney impairment (CKI) was defined as CrCl < 60 mL/min. Operative variables included aortic cross-clamp time (CCT), CPB duration, and number of grafts. The number of RBC units transfused was also recorded. Because of the high correlation between CCT and CPB duration (Pearson r = 0.876; Spearman ρ = 0.891; *p* < 0.001), only CPB duration was included in the primary models. EuroSCORE II was evaluated separately. Infections were classified according to their anatomical sites. Smoking status and number of grafts were used only in post hoc preoperative/operative sensitivity models, whereas RBC transfusion (yes/no and recorded number of units) and cerebrovascular events were used in expanded hospitalization-level sensitivity models.

The primary outcome was the cumulative number of postoperative hospital days among patients who survived to 30 days. Counting began on the date of surgery and included the index hospitalization and the total number of recorded readmission days within 30 days after surgery; time spent outside the hospital was not included. Accordingly, the primary estimand was survivor-specific, and conditioning on survival may introduce selection related to baseline risk and postoperative complications. Patients transferred to another institution for whom complete follow-up information could not be obtained were excluded because of incomplete records. In the whole-cohort sensitivity analysis, the recorded interval from surgery to death was retained for patients who died. This analysis assessed the robustness of the findings to the inclusion of patients who died but did not model discharge and death as competing events.

Because the median LOS among 30-day survivors was 7 days, LOS > 7 days was defined as a data-derived, center-specific complementary binary outcome for prolonged hospitalization.

Other postoperative outcomes included POAF, postoperative infection, new dialysis, prolonged mechanical ventilation, postoperative vasoactive/inotropic support, use of an intra-aortic balloon pump (IABP) or extracorporeal membrane oxygenation (ECMO), cerebrovascular events, and 30-day mortality.

POAF was defined as new-onset atrial fibrillation documented by rhythm monitoring or 12-lead electrocardiography during the index hospitalization. New dialysis was defined as initiation of continuous renal replacement therapy or intermittent hemodialysis in patients not receiving dialysis preoperatively. Prolonged mechanical ventilation was defined as continuous invasive ventilation lasting >24 h after surgery. Postoperative vasoactive/inotropic support included dopamine, dobutamine, epinephrine, or norepinephrine, with no minimum duration requirement. IABP and ECMO use referred to any postoperative use. A cerebrovascular event was defined as a documented postoperative cerebrovascular event. Thirty-day mortality was defined as death from any cause within 30 days after surgery.

Postoperative infection was defined as any infection documented by a clinician during the index hospitalization and ascertained from the medical record, including culture results when available. A uniform Centers for Disease Control and Prevention (CDC) or Society of Thoracic Surgeons (STS) surveillance definition was not applied. Infections were classified as superficial sternal wound infection, saphenous vein harvest-site infection, pneumonia, mediastinitis, or combinations of these conditions. A total of 255 infections were evaluated using this classification.

Because mediastinitis included sternal involvement, this involvement was not counted separately. Superficial sternal infection occurring in the absence of mediastinitis was classified as a separate category. Sepsis was assigned to the documented primary site of infection. Because urinary tract infection could not be reliably identified as a distinct category during broad-spectrum antibiotic therapy, no independent urinary tract infection category was created. Similarly, an independent bloodstream infection category was not defined.

Postoperative events were coded as binary variables according to whether they occurred at any time during the index hospitalization. Exact timing data were unavailable for infection, the first episode of POAF, dialysis initiation, and the course of mechanical ventilation. Consequently, the temporal sequence of these events in relation to accumulated hospital days and prespecified clinical time points could not be determined.

### 2.3. Statistical Analysis

The primary analysis modeled postoperative hospital days among 30-day survivors using negative binomial regression with a log link. This method was selected because the LOS distribution was right-skewed and overdispersed and provided a better fit than Poisson regression (Supplementary Table S3). For uncertainty estimation in the negative binomial models, an HC0 sandwich covariance estimator conditional on the estimated dispersion parameter was used. LOS > 7 days was examined using complementary multivariable logistic regression in the same survivor cohort. The postoperative event–LOS models and the models for POAF, infection, and 30-day mortality were secondary analyses; the infection-site analysis was exploratory, and the EuroSCORE II evaluation was auxiliary.

Normally distributed continuous variables are presented as mean ± standard deviation, skewed variables as median [interquartile range], and categorical variables as *n* (%). Continuous variables were compared using Welch’s *t*-test or the Mann–Whitney U test, whereas categorical variables were compared using Pearson’s chi-square test or Fisher’s exact test.

Both primary LOS models used the same adjustment set, comprising age, sex, BMI, diabetes, hypertension, COPD, LVEF, CKI, Hb, and CPB duration. Covariates were prespecified based on clinical relevance and the CABG literature; neither univariable *p* values nor stepwise selection procedures were used. Results are reported as incidence rate ratios (IRRs) or adjusted odds ratios (aORs) with 95% confidence intervals. Continuous variables were scaled per 10-year increase in age, per 5 kg/m^2^ increase in BMI, per 5-percentage-point decrease in LVEF, per 1 g/dL decrease in Hb, and per 30-min increase in CPB duration.

The primary count model was repeated using log-linear regression, median (τ = 0.50) and 75th-percentile (τ = 0.75) quantile regression, and zero-truncated negative binomial regression with the same covariates. The primary count and LOS > 7 days models were also fitted in the entire cohort, including patients who died. In both survivor-cohort models, age, BMI, LVEF, Hb, and CPB duration were evaluated separately using restricted cubic splines with 3 degrees of freedom and compared with their corresponding linear terms using likelihood-ratio tests; a joint test of nonlinearity was also performed. Year of surgery was additionally entered into both models as a categorical variable, with 2020 as the reference year.

In the secondary LOS models, infection, POAF, new dialysis, and prolonged mechanical ventilation were entered simultaneously as separate terms together with the prespecified covariates. These binary event indicators, which encompassed the entire hospitalization, evaluated only adjusted contemporaneous associations at the hospitalization level and did not assess incremental prediction at a specific time point. Because event-onset times were unavailable, landmark and time-dependent covariate analyses could not be performed. As new dialysis occurred in only nine survivors, the LOS > 7 days model was also repeated using Firth logistic regression.

In the exploratory infection-site analysis, the binary infection term was replaced with five groups: no infection, superficial wound infection only, pneumonia only, mediastinitis without an additional extrasternal focus, and multisite infection. The negative binomial model retained the other baseline, operative, and postoperative covariates; year of surgery was added to the sensitivity model.

The post hoc sensitivity analyses did not alter the prespecified primary model. Smoking status and number of grafts were added to the preoperative/operative LOS models. The expanded hospitalization-level models additionally included any RBC transfusion (recorded number of units > 0; yes/no) and cerebrovascular events. In a separate dose-based sensitivity analysis, the binary transfusion term was replaced with the recorded number of RBC units transfused, scaled per two-unit increase. Because the exact timing of RBC transfusion and cerebrovascular events was unknown, the coefficients for these variables were used solely to assess model stability and were not interpreted causally.

Separate multivariable logistic regression models for POAF and postoperative infection each included nine preoperative variables; CPB duration was added in the sensitivity models. Because only 44 deaths occurred, the mortality analyses were conducted on an exploratory basis, and Firth penalized logistic regression was used to reduce small-sample bias. The 10-parameter multivariable model was retained solely for descriptive comparison. The parsimonious two-variable model included log_2_-transformed EuroSCORE II and CPB duration. Discrimination, Brier score, calibration intercept, and calibration slope were internally validated using 2000 bootstrap resamples and corrected for optimism. Continuous year of surgery was added to the sensitivity model. The association between POAF and mortality was examined sequentially using an unadjusted model, a model adjusted for baseline and operative factors, and a model additionally adjusted for major postoperative events.

In the auxiliary score analysis, EuroSCORE II was log_2_-transformed so that each unit increase represented a twofold increase in predicted mortality risk. Its local performance for 30-day mortality was evaluated using the area under the receiver operating characteristic curve (AUC), observed-to-expected mortality ratio, calibration intercept and slope, and Brier score, together with their 95% confidence intervals. The DeLong method was used for the AUC, and bootstrap resampling with 5000 samples was used for the Brier score. This exploratory single-center assessment was not considered model development or external validation. Post hoc exploratory analyses evaluated the association and local discriminatory performance of EuroSCORE II for postoperative infection, the center-specific LOS > 7 days outcome, and postoperative cerebrovascular events. EuroSCORE II was modeled using the log_2_ transformation, and the results were expressed as odds ratios (ORs) per twofold increase in the score. Apparent discrimination was evaluated using the AUC with DeLong 95% confidence intervals. Analyses of infection and cerebrovascular events were conducted in the entire cohort, whereas LOS analyses were restricted to patients who survived to 30 days. Because EuroSCORE II was developed for mortality, calibration measures and observed-to-expected ratios were not calculated for the other outcomes, and no data-driven threshold was derived.

All tests were two-sided, and *p* < 0.05 was considered statistically significant. Analyses were performed using IBM SPSS Statistics for Windows, version 24.0 (IBM Corp., Armonk, NY, USA), R version 4.6.0 (R Foundation for Statistical Computing, Vienna, Austria), and Python version 3.12 (Python Software Foundation, Wilmington, DE, USA) with statsmodels version 0.15.0 (Statsmodels Developers; https://www.statsmodels.org/). Generative AI was used only for English-language editing and preparation of the graphical abstract; all outputs were reviewed by the authors, and the study data and analyses were independently verified.

## 3. Results

### 3.1. Cohort and Clinical Outcomes

Among the 1493 patients, 44 (2.95%) died within 30 days after surgery; all deaths occurred during the index hospitalization. Of the 1449 patients who survived to 30 days, 406 (28.0%) had an LOS > 7 days. The median ICU and postoperative hospital lengths of stay were 1 [1–2] and 7 [6–8] days, respectively. In the entire cohort, POAF occurred in 278 patients (18.6%), postoperative infection in 255 (17.1%), and cerebrovascular events in 37 (2.5%).

Among survivors, patients with an LOS > 7 days were older and had higher prevalences of diabetes, hypertension, COPD, and smoking. They also had lower preoperative Hb levels, longer CCT and CPB durations, and a greater number of grafts. EuroSCORE II was also higher in the LOS > 7 days group (LOS > 7 days: 1.56 [1.07–2.46]; LOS ≤ 7 days: 1.33 [0.92–2.11]; *p* < 0.001). A statistically significant but weak positive correlation was observed between EuroSCORE II and cumulative LOS (Spearman ρ = 0.149; *p* < 0.001; Table 1).

Among survivors, postoperative events were more frequent in the LOS > 7 days group. POAF occurred in 31.0% versus 12.7%, infection in 29.8% versus 10.6%, and prolonged mechanical ventilation in 17.7% versus 2.9% (all *p* < 0.001). The rate of cerebrovascular events was also higher in this group (3.2% versus 0.9%; *p* = 0.001), whereas the proportion of patients receiving RBC transfusion did not differ between the groups (*p* = 0.422; Table 2).

### 3.2. Primary Analysis: Preoperative and Operative Factors Associated with Hospital Length of Stay

**Figure 2:**
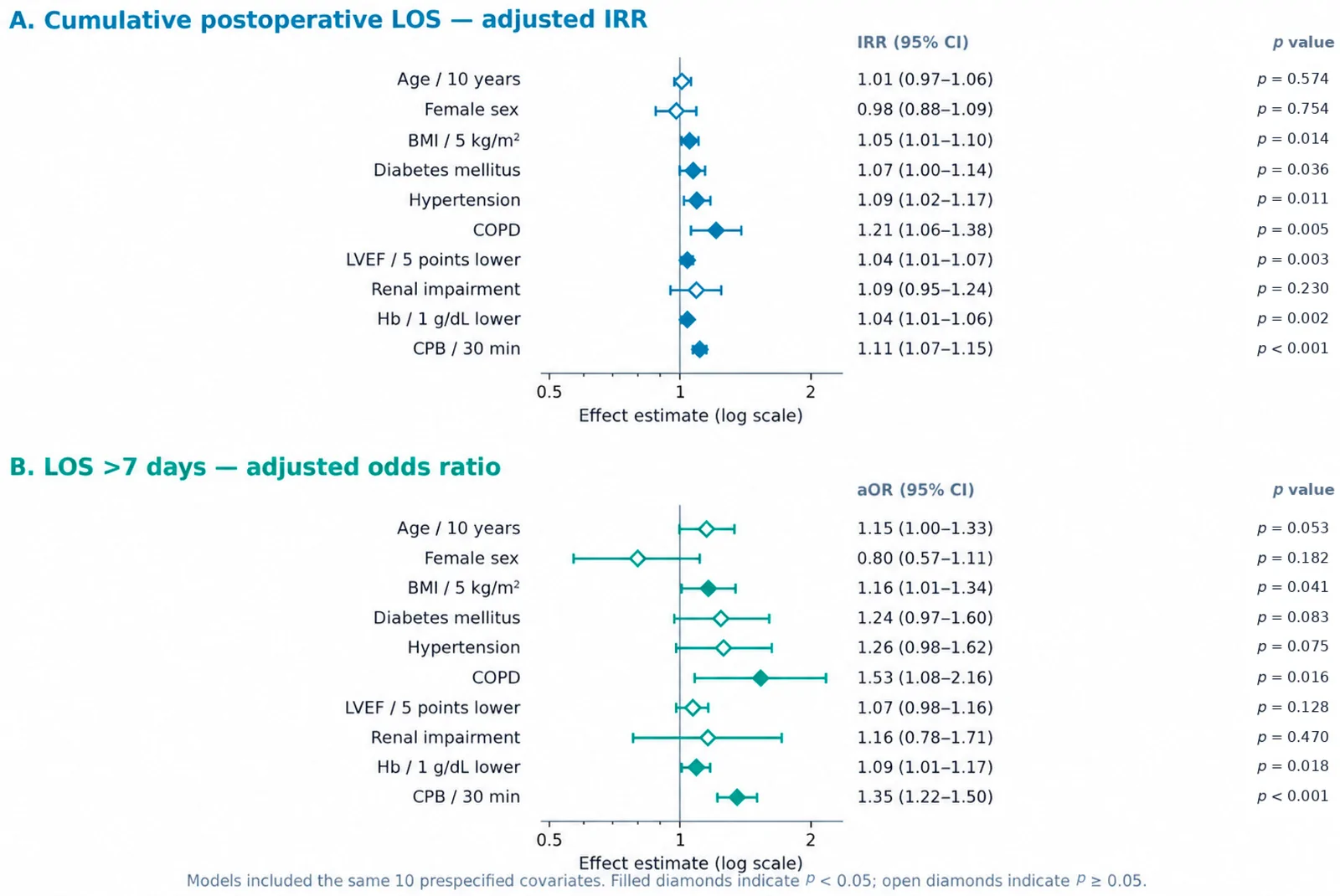
Analysis of factors associated with postoperative hospital length of stay among 30-day survivors. (**A**) IRRs from the negative binomial model of cumulative postoperative hospital days; (**B**) aORs from the logistic regression model for LOS > 7 days (n = 1449; LOS > 7 days, n = 406). Filled diamonds indicate *p* < 0.05, open diamonds indicate *p* ≥ 0.05, and the vertical line indicates the null value (1.0). An IRR or aOR >1 indicates an association with longer LOS. Continuous variables were scaled per 10-year increase in age, per 5 kg/m^2^ increase in BMI, per 5-percentage-point decrease in LVEF, per 1 g/dL decrease in hemoglobin, and per 30-min increase in CPB duration. aOR, adjusted odds ratio; BMI, body mass index; CPB, cardiopulmonary bypass; IRR, incidence rate ratio; LOS, length of stay; LVEF, left ventricular ejection fraction.

In the primary negative binomial model, longer LOS was associated with higher BMI (IRR per 5 kg/m^2^ increase, 1.05), diabetes (IRR, 1.07), hypertension (IRR, 1.09), COPD (IRR, 1.21), lower LVEF (IRR per 5-percentage-point decrease, 1.04), lower Hb (IRR per 1 g/dL decrease, 1.04), and longer CPB duration (IRR per 30-min increase, 1.11). After adjustment, age, sex, and CKI were not associated with LOS. In the complementary logistic regression model, higher BMI, COPD, lower Hb, and longer CPB duration were associated with LOS > 7 days (Table 3, Panel A; Figure 2).

The direction of the associations was preserved in the log-linear and zero-truncated models and in the entire-cohort analysis that included patients who died. In the quantile regression analyses, CPB duration was associated with both median and 75th-percentile LOS, whereas the association with COPD was more pronounced at the 75th percentile. In the spline analysis, the only signal of nonlinearity was observed for Hb (*p* = 0.011), and this signal attenuated after adjustment for year of surgery (*p* = 0.114); the other individual and joint tests showed no clear evidence of nonlinearity. Linear terms were therefore retained, and the Hb IRR was interpreted as the average association across the observed range (Supplementary Tables S1–S3; Supplementary Figure S1).

Year of surgery was associated with both LOS outcomes (overall *p* < 0.001). Categorical adjustment for year of surgery changed the primary IRRs in the count model by no more than 3.7%. The association with diabetes was attenuated (IRR, 1.06; 95% CI, 0.99–1.14; *p* = 0.093), whereas the other primary associations were preserved. The LOS > 7 days model was more sensitive to calendar time, consistent with the marked decrease in the proportion of patients with LOS > 7 days after 2022 (Supplementary Table S8).

Adding smoking status and number of grafts changed the primary IRRs by no more than 2.4%. Smoking was not associated with LOS. Although the number of grafts showed a small inverse association in the count model, this finding was not replicated in the LOS > 7 days model. Because CPB duration was also included in the model, the coefficient for the number of grafts was not interpreted as protective (Table 3, Panel B).

### 3.3. Secondary Outcome Analyses: POAF and Postoperative Infection

**Figure 3:**
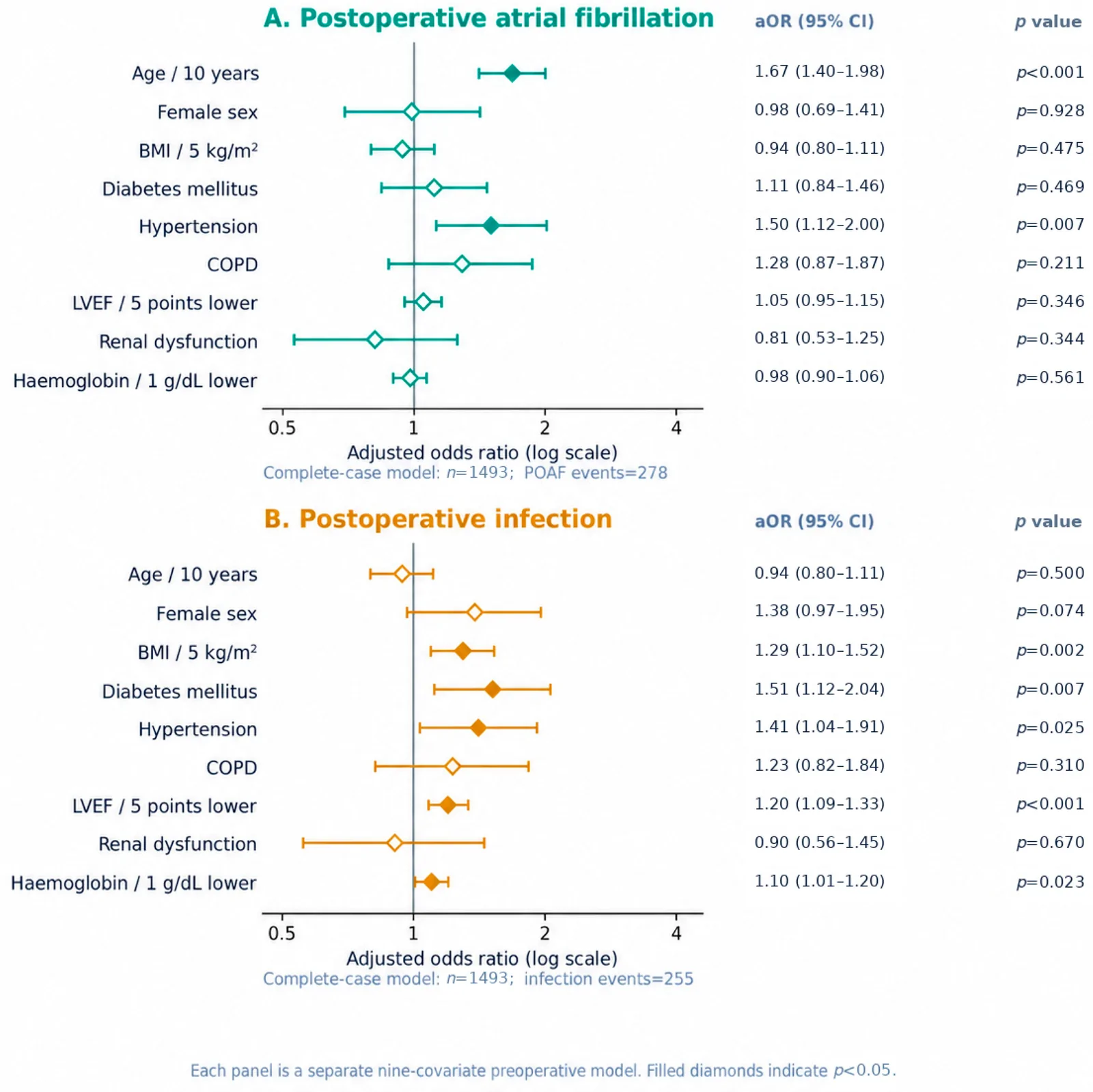
Adjusted associations of preoperative characteristics with postoperative atrial fibrillation and infection. (**A**) Postoperative atrial fibrillation (278 events) and (**B**) postoperative infection (255 events). Filled diamonds indicate *p* < 0.05, open diamonds indicate *p* ≥ 0.05, and the vertical line indicates the null value (aOR = 1.0). Continuous variables were scaled per 10-year increase in age, per 5 kg/m^2^ increase in BMI, per 5-percentage-point decrease in LVEF, and per 1 g/dL decrease in hemoglobin. Perioperative sensitivity models additionally including CPB duration are presented in Supplementary Table S4. aOR, adjusted odds ratio; BMI, body mass index; CPB, cardiopulmonary bypass; LVEF, left ventricular ejection fraction.

Older age (aOR per 10-year increase, 1.67) and hypertension (aOR, 1.50) were associated with POAF after adjustment. Both associations persisted after CPB duration was added to the model, and longer CPB duration was also associated with POAF (aOR per 30-min increase, 1.14; Table 4; Figure 3A; Supplementary Table S4, Panel A, for the CPB sensitivity analysis).

Higher BMI (aOR, 1.29), diabetes (aOR, 1.51), hypertension (aOR, 1.41), lower LVEF (aOR per 5-percentage-point decrease, 1.20), and lower Hb (aOR per 1 g/dL decrease, 1.10) were associated with postoperative infection after adjustment. These findings persisted after CPB duration was added to the model (Table 4; Figure 3B; Supplementary Table S4, Panel A, for the CPB sensitivity analysis).

### 3.4. Secondary LOS Analysis: Contemporaneous Associations of Postoperative Events with LOS

**Figure 4:**
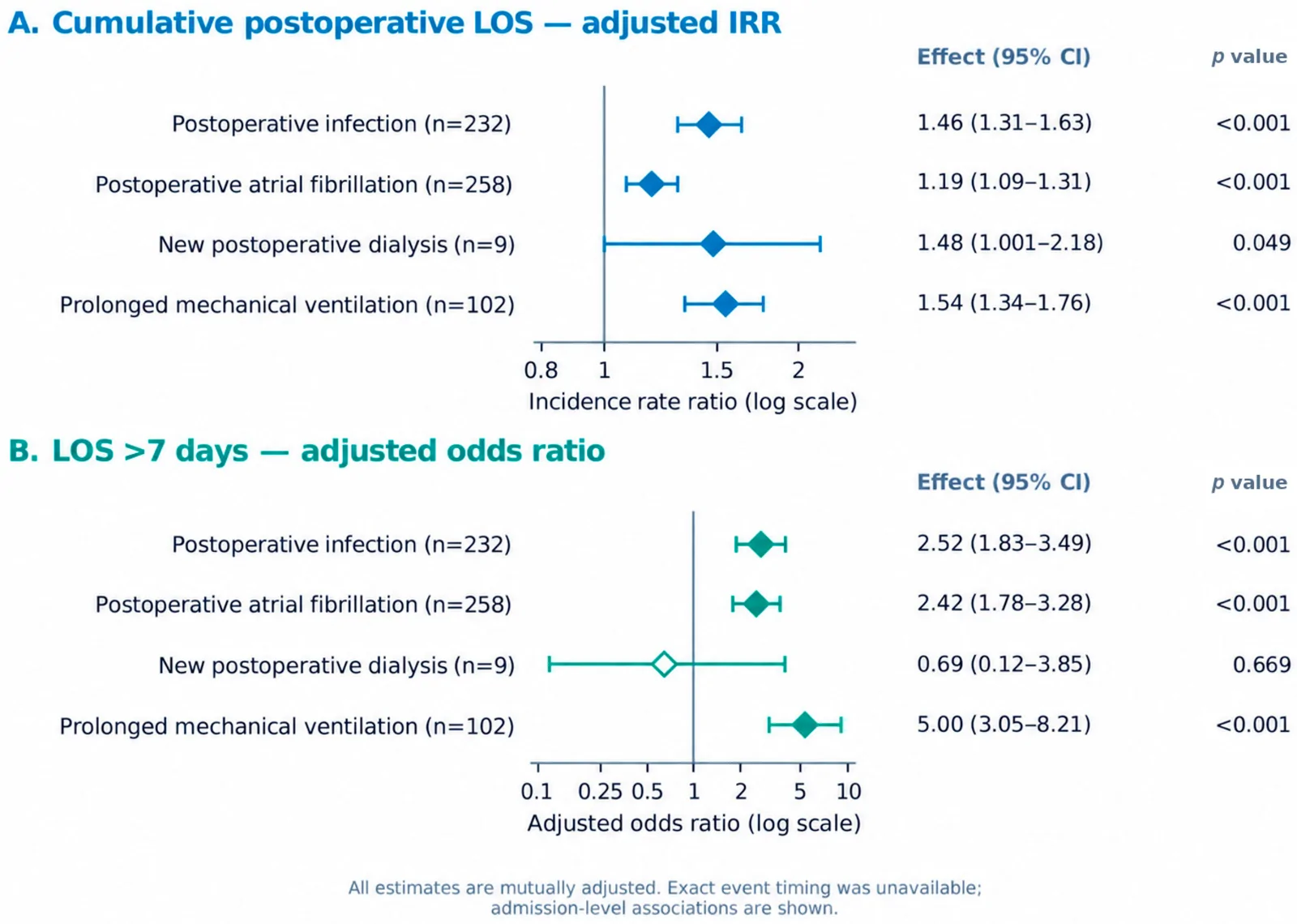
Adjusted contemporaneous associations of postoperative events with hospital length of stay. (**A**) IRRs from the negative binomial model for cumulative postoperative hospital days among 1449 patients who survived to 30 days; (**B**) aORs from the standard logistic regression model for LOS > 7 days. Models simultaneously included age, sex, BMI, diabetes, hypertension, COPD, LVEF, CKI, hemoglobin, CPB duration, and the four postoperative events shown. Filled diamonds indicate *p* < 0.05, open diamonds indicate *p* ≥ 0.05, and the vertical line indicates the null value (1.0). New dialysis occurred in only nine survivors; the estimates are imprecise. Because event timing was unavailable, results represent hospitalization-level associations rather than temporal, predictive, or causal effects. aOR, adjusted odds ratio; BMI, body mass index; CKI, chronic kidney impairment; COPD, chronic obstructive pulmonary disease; CPB, cardiopulmonary bypass; IRR, incidence rate ratio; LOS, length of stay; LVEF, left ventricular ejection fraction.

In the secondary model adjusted for the prespecified preoperative and operative variables, infection (IRR, 1.46), POAF (IRR, 1.19), and prolonged mechanical ventilation (IRR, 1.54) were associated with longer LOS. The corresponding adjusted odds ratios for LOS > 7 days were 2.52, 2.42, and 5.00, respectively. Because new dialysis occurred in only nine patients who survived to 30 days, the estimate for this event was imprecise (Figure 4). As events were recorded as present or absent over the entire index hospitalization, these findings represent contemporaneous hospitalization-level associations rather than temporal or causal effects.

In the post hoc sensitivity model incorporating additional variables, the estimates for infection (IRR, 1.45), POAF (IRR, 1.21), and prolonged mechanical ventilation (IRR, 1.52) remained largely unchanged (Table 3, Panel B).

**Figure 5:**
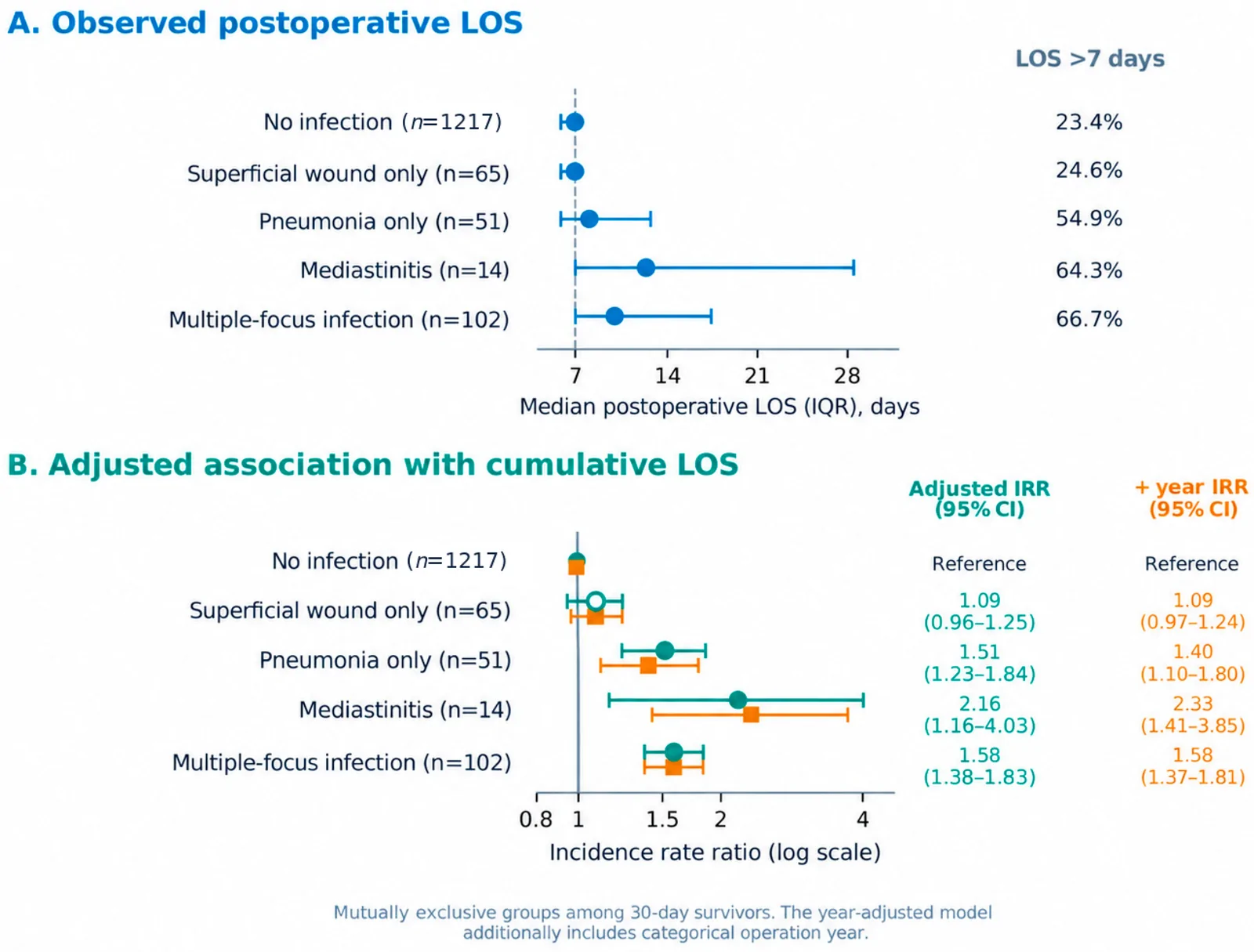
Observed length of stay and adjusted associations according to postoperative infection site. (**A**) Median postoperative LOS and interquartile range by infection group among 30-day survivors; (**B**) IRRs from exploratory negative binomial models using no infection as the reference category. Circles indicate the model adjusted for the prespecified covariates and other postoperative events; squares indicate the sensitivity model additionally adjusted for categorical year of surgery. Infection groups were mutually exclusive. The mediastinitis group included only 14 survivors; the estimate is imprecise. Results represent contemporaneous hospitalization-level associations. IRR, incidence rate ratio; LOS, length of stay.

Of the 255 documented infections, 73 were superficial wound infections only, 56 were pneumonia, 15 were mediastinitis without another extrasternal focus, and 111 were multisite infections. In the exploratory analysis, superficial wound infection alone was not associated with LOS (IRR, 1.09; 95% CI, 0.96–1.25; *p* = 0.179). In contrast, pneumonia (IRR, 1.51), mediastinitis (IRR, 2.16), and multisite infection (IRR, 1.58) were associated with longer LOS. Adjustment for year of surgery did not materially alter this pattern (Figure 5B; the detailed distribution of infection-site groups is presented in Supplementary Table S5). These findings were also interpreted as exploratory hospitalization-level associations. In an alternative post hoc sensitivity model in which baseline risk was summarized using EuroSCORE II, infection remained associated with both cumulative LOS (IRR, 1.52; 95% CI, 1.35–1.71) and LOS > 7 days (aOR, 2.69; 95% CI, 1.97–3.68; *p* < 0.001 for both).

### 3.5. Exploratory Analysis of 30-Day Mortality

Because only 44 deaths occurred, all mortality models were considered exploratory. In the parsimonious Firth model, each twofold increase in EuroSCORE II was associated with higher odds of mortality (OR, 2.01; 95% CI, 1.55–2.62). Similarly, each 30-min increase in CPB duration was associated with higher odds of mortality (OR, 1.78; 95% CI, 1.49–2.14). The apparent AUC was 0.801, and the optimism-corrected AUC was 0.799 (95% bootstrap CI, 0.737–0.868). The optimism-corrected Brier score was 0.0268 (0.0195–0.0338), the calibration intercept was −0.008 (−0.309 to 0.341), and the calibration slope was 0.992 (0.777–1.268; Supplementary Table S6, Sections B–D).

The full 10-parameter Firth model had 4.4 deaths per parameter and was retained only as an exploratory descriptive analysis (Supplementary Figure S2B). The individual coefficients from this model were not interpreted as stable confirmatory estimates. In the parsimonious sensitivity model additionally including continuous year of surgery, the odds ratios for EuroSCORE II and CPB duration changed by 7.3% and 2.5%, respectively. A later year of surgery was associated with lower odds of mortality (OR per year, 0.78; 95% CI, 0.63–0.95).

Thirty-day mortality was 1.98% among patients without POAF and 7.19% among those with POAF (unadjusted risk ratio, 3.64; Firth OR, 3.86). POAF remained associated with mortality after adjustment for baseline factors (aOR, 2.97). However, this association attenuated after postoperative infection, dialysis, and prolonged mechanical ventilation were added to the model (aOR, 1.76; *p* = 0.172), a finding consistent with overlapping postoperative complication burden (Supplementary Table S4, Panel B).

### 3.6. Auxiliary Analysis: Local Performance and Exploratory Associations of EuroSCORE II

In the local evaluation of the fixed score, each twofold increase in EuroSCORE II was associated with higher odds of 30-day mortality (OR, 2.10; 95% CI, 1.63–2.72; *p* < 0.001). EuroSCORE II predicted 30.57 deaths, compared with 44 observed deaths, yielding an observed-to-expected mortality ratio of 1.44 (95% CI, 1.05–1.93). Apparent discrimination was moderate (AUC, 0.694; 95% CI, 0.613–0.775). The calibration intercept was 0.385 (95% CI, 0.080–0.691), the calibration slope was 1.008 (0.663–1.352), and the Brier score was 0.0278 (bootstrap 95% CI, 0.0199–0.0359; Supplementary Figure S2A; Supplementary Table S6, Section A).

In the post hoc analyses, each twofold increase in EuroSCORE II was associated with postoperative infection (OR, 1.40; 95% CI, 1.22–1.61), the center-specific LOS > 7 days outcome (OR, 1.39; 95% CI, 1.22–1.58), and postoperative cerebrovascular events (OR, 1.76; 95% CI, 1.33–2.34; *p* < 0.001 for all). However, discrimination was poor for infection (AUC, 0.580) and LOS > 7 days (AUC, 0.569). Although the AUC for cerebrovascular events was 0.654, the estimate was imprecise because only 37 events occurred (Supplementary Table S6, Section E). These findings do not support the use of EuroSCORE II as a stand-alone clinical classification tool for these outcomes.

## 4. Discussion

The hospital stay following an elective isolated CABG was not attributable to a single factor. Higher BMI, diabetes, hypertension, COPD, lower LVEF and Hb, and longer CPB duration were associated with more postoperative hospital days among patients who survived to 30 days. Infection, POAF, and prolonged mechanical ventilation were postoperative correlates accompanying longer hospitalization. This distinction is important: preoperative characteristics reflect baseline patient reserve, CPB duration reflects operative burden, and postoperative events reflect the observed recovery course and burden of care.

Modeling LOS primarily as a count outcome preserved the information contained in the number of hospital days. The LOS > 7 days analysis provided a complementary representation that was easier to interpret clinically. The findings were broadly consistent across the alternative count, log-linear, quantile, and entire-cohort analyses. Adjustment for year of surgery changed the primary count-model estimates only modestly, although the association with diabetes was attenuated. In contrast, the LOS > 7 days outcome was more sensitive to changes across calendar years. The decrease in hospitalizations exceeding this threshold, particularly after 2022, suggests that this outcome reflects local discharge practices as well as clinical recovery. Therefore, the primary inferences should be based on cumulative hospital days, whereas the >7-day threshold should be interpreted as a center-specific complementary measure [1,10].

Among the baseline characteristics, COPD showed the strongest relative association with LOS. This association also persisted for the LOS > 7 days outcome. Reduced respiratory reserve may delay postoperative mobilization and weaning from respiratory support. However, pulmonary function tests and detailed clinical measures of COPD severity were not recorded. Consequently, a potential dose–response relationship between disease severity and LOS could not be evaluated. Previous studies have also associated COPD and respiratory complications with prolonged hospitalization after CABG [7,8,9].

Preoperative CKI was not associated with LOS after adjustment. This finding does not imply that renal function is clinically unimportant. Restricting the cohort to elective patients may have narrowed the distribution of renal risk. Furthermore, dichotomizing renal function as CrCl < 60 mL/min may have obscured a graded association. Overlap with other comorbidities may also have attenuated the adjusted association [16].

Higher BMI was associated with longer LOS and postoperative infection but not with POAF or mortality. This difference suggests that the association between BMI and postoperative recovery may vary according to the outcome considered. Diabetes and hypertension were also associated with modest increases in hospital days; however, these associations were not consistent for the LOS > 7 days outcome.

Lower preoperative Hb was associated with longer LOS. Adjustment for year of surgery did not eliminate this association but weakened the evidence of a nonlinear pattern. Therefore, the reported linear IRR should be interpreted as the average association across the observed Hb range rather than as a constant biological effect at all Hb levels. In a cohort of 53,856 patients undergoing elective CABG, anemia was associated with mortality, dialysis, transfusion, and prolonged hospitalization [17]. Current recommendations also support the preoperative evaluation of anemia and iron deficiency [18].

The inclusion of only elective isolated CABG cases may have resulted in underrepresentation of patients with severe anemia or those requiring preoperative treatment. This may have narrowed the Hb distribution and attenuated the magnitude of the association. Moreover, data on iron status, the cause of anemia, and the exact timing of RBC transfusion were unavailable. Consequently, the effect of lower Hb could not be fully separated from those of comorbidities, perioperative blood loss, and RBC transfusion. The findings support lower Hb as a marker of reduced hematological reserve rather than establishing anemia as a causal factor.

Longer CPB duration was consistently associated with LOS, POAF, infection, and 30-day mortality. Previous studies have also associated prolonged CPB exposure with adverse outcomes after CABG [19]. CPB duration may reflect both procedural complexity and the accompanying inflammatory, hemostatic, and perfusion burdens. The association between CPB duration and LOS persisted after the number of grafts was added to the model. In contrast, the small inverse coefficient for the number of grafts should not be interpreted as a protective effect. Both variables represent correlated aspects of operative burden. Similarly, CPB duration should be regarded as a marker of operative burden rather than as a stand-alone quality indicator.

Postoperative events yielded the numerically largest hospitalization-level estimates. Infection, POAF, and prolonged mechanical ventilation were associated with 46%, 19%, and 54% longer expected LOS, respectively. These associations were also pronounced for the complementary >7-day outcome, particularly for prolonged ventilation. The infection-site analysis demonstrated heterogeneity: after adjustment, superficial wound infection alone was not associated with LOS, whereas pneumonia, mediastinitis, and multisite infection were associated with longer LOS. Because only 14 survivors had mediastinitis, the corresponding estimate was imprecise.

These findings are consistent with reports linking arrhythmia, infection, and respiratory failure to hospital stay [2,5,7,8,9]. However, postoperative events and LOS were recorded within the same hospitalization, and exact event times were not retained. This limitation makes the direction of the association particularly difficult to determine for infection: a longer hospitalization may provide more time for an infection to develop or be detected, whereas infection may itself delay discharge. Although POAF and prolonged ventilation would clinically be expected to occur during the early postoperative period, the absence of timestamps precluded evaluation of their prospective predictive value at a defined clinical time point. The estimates should therefore be interpreted as contemporaneous hospitalization-level associations rather than causal effects.

POAF developed in 18.6% of patients, a rate slightly lower than the approximately 25% reported after cardiac surgery [11]. The inclusion of only elective isolated CABG cases and differences in age, monitoring practices, or event definitions may explain this difference. Older age, hypertension, and longer CPB duration were associated with POAF. POAF remained associated with longer LOS after adjustment for the other measured postoperative events. Its association with mortality persisted after adjustment for baseline factors but attenuated after infection, dialysis, and prolonged ventilation were added to the model. This pattern may reflect shared illness severity, overlapping complications, or overadjustment; it does not demonstrate that POAF causes prolonged hospitalization or mortality [13,14,15].

The mortality findings should be interpreted in light of the limited number of events. Because only 44 deaths occurred, all mortality analyses were exploratory. The two-parameter model containing EuroSCORE II and CPB duration had an optimism-corrected AUC of 0.799. Although bootstrap correction indicated little optimism in the apparent performance, it provides no information regarding external validity. Furthermore, although the Firth method reduces small-sample bias, it does not eliminate the risk of overfitting in a 10-parameter model with 44 events. The coefficients from the expanded model should therefore be considered descriptive only. The adjusted associations of age, LVEF, CPB duration, and hemoglobin with 30-day mortality were broadly consistent with previous reports [16,17,20,21]; however, the limited event count also restricts the precision of these estimates. When evaluated alone, EuroSCORE II showed moderate discrimination (AUC, 0.694) and underestimated mortality (observed-to-expected ratio, 1.44). These findings describe local model performance and should not be generalized without validation in an independent cohort [22,23,24].

The associations of EuroSCORE II with outcomes other than mortality were also examined post hoc. Each twofold increase in the score was associated with postoperative infection (OR, 1.40), LOS > 7 days (OR, 1.39), and cerebrovascular events (OR, 1.76). However, discrimination was poor for infection and LOS > 7 days (AUC, 0.580 and 0.569, respectively). Although the AUC was higher for cerebrovascular events, discrimination remained moderate and was based on only 37 events (AUC, 0.654; 95% CI, 0.560–0.747). These findings therefore do not support using EuroSCORE II as a stand-alone prediction tool for these outcomes. In contrast, infection remained associated with cumulative LOS after adjustment for EuroSCORE II, CPB duration, POAF, new dialysis, and prolonged ventilation (IRR, 1.52; 95% CI, 1.35–1.71). This finding suggests that the infection–LOS association was not fully explained by baseline risk as summarized by EuroSCORE II. However, because the time of infection onset was unknown, this association should be considered contemporaneous and hospitalization-level rather than temporal or causal.

LOS may be relevant to planning postoperative care; however, direct costs, staffing requirements, bed availability, and the use of discharge resources were not measured. Consequently, interpretations regarding resource planning are hypothesis-generating and require prospective evaluation.

### Strengths and Limitations

The principal strengths of this study include its large, homogeneous cohort of patients undergoing elective isolated CABG and complete data for the analysis variables. Derived measurements were recalculated and verified. Modeling LOS as a count outcome prevented the loss of information that would result from binary classification. The functional forms of continuous variables were examined, and the findings were tested using alternative models and adjustment for year of surgery. The mortality model was kept parsimonious and internally validated using bootstrap resampling. EuroSCORE II was evaluated separately from the primary LOS models.

Nevertheless, the retrospective, single-center design precludes causal inference and limits generalizability. Excluding emergency procedures reduced the clinical diversity of the cohort. Because standardized covariates were unavailable for the excluded records, the potential effect of selection could not be quantified. The exact onset times of postoperative events were also unknown; therefore, their timing relative to accrued hospital days and their predictive value after a defined clinical time point could not be determined. For infection in particular, the possibility that a longer hospitalization preceded the event and increased the opportunity for its detection could not be excluded. Because landmark and time-dependent covariate analyses could not be performed, the findings concerning postoperative events demonstrate only contemporaneous hospitalization-level associations.

The infection classification was based on retrospective records, and some subgroups were small. Sternal involvement accompanying mediastinitis could not be evaluated separately, and urinary tract and bloodstream infections could not be reliably distinguished as independent categories. Although data on smoking status, number of grafts, RBC transfusion, and cerebrovascular events were complete, misclassification remains possible. The transfused product was red blood cells, and the number of units was recorded; however, exact transfusion times were unavailable. Preoperative atrial fibrillation, POAF burden, frailty, nutritional status, albumin, left atrial size, medication use, and reoperation data were also not recorded. In addition, ICU capacity, access to rehabilitation, discharge destination, caregiver support, and local discharge policies were not measured. These clinical, organizational, and social factors may affect LOS independently of physiological recovery. Residual confounding therefore remains possible, and adjusted estimates should be interpreted as conditional associations rather than causal effects.

Restricting the primary LOS analysis to patients who survived to 30 days may have introduced survivor-selection bias. Similar findings in the entire-cohort analyses supported the robustness of the results to the inclusion of patients who died; however, these analyses could not recover hospital days that were unobservable after death. Because exact discharge and readmission dates were unavailable, a competing-risk analysis of time to first discharge could not be performed. The LOS > 7 days threshold was data-derived, center-specific, and sensitive to year of surgery.

The occurrence of only 44 deaths limited the reliability of the mortality models. Although Firth regression reduces small-sample coefficient bias, it does not eliminate the risk of overfitting, and bootstrap internal validation cannot substitute for external validation. Accordingly, the expanded mortality model and all auxiliary analyses involving EuroSCORE II should be considered exploratory. In particular, the nonmortality analyses do not validate EuroSCORE II as a prediction tool for infection, prolonged LOS, or cerebrovascular events.

## 5. Conclusions

LOS following elective isolated CABG is a multidimensional clinical outcome that reflects preoperative patient reserve, operative burden, and the postoperative recovery course. Among patients who survived to 30 days, higher BMI, diabetes, hypertension, COPD, lower LVEF and Hb, and longer CPB duration were associated with increased cumulative hospital days. The most consistent associations were observed for COPD and CPB duration. POAF and prolonged mechanical ventilation were associated with a more complex postoperative course and longer hospitalization. The association between infection and LOS persisted after adjustment for EuroSCORE II, CPB duration, and the other major postoperative events. However, the unknown timing of infection onset precluded determination of the temporal direction of this association. In the exploratory mortality analyses, EuroSCORE II and CPB duration were associated with 30-day mortality; EuroSCORE II alone showed moderate local discrimination and underestimated observed mortality. These findings highlight the importance of jointly considering preoperative respiratory, metabolic, and hematological reserve, operative burden, and the postoperative complication burden that actually occurred when evaluating hospital resource utilization. Prospective, multicenter studies are required to validate these associations before they can be translated into clinical decision-making.

## Figures and Tables

**Table 1:** Baseline and operative characteristics of the cohort and of 30-day survivors according to LOS category.

Variable	Entire Cohort *n* = 1493	LOS ≤ 7 Days *n* = 1043	LOS > 7 Days *n* = 406	*p*
**Demographic and preoperative characteristics**				
Female sex	283 (19.0%)	192 (18.4%)	78 (19.2%)	0.724
Age, years	61.2 ± 9.1	60.6 ± 8.9	62.3 ± 9.3	0.002
Body mass index, kg/m^2^	28.3 ± 4.3	28.2 ± 4.2	28.6 ± 4.5	0.062
Documented smoking	274 (18.4%)	170 (16.3%)	88 (21.7%)	0.016
Diabetes mellitus	857 (57.4%)	572 (54.8%)	255 (62.8%)	0.006
Hypertension	914 (61.2%)	609 (58.4%)	271 (66.7%)	0.003
COPD	178 (11.9%)	106 (10.2%)	63 (15.5%)	0.004
Preoperative LVEF, %	45.0 [40.0–50.0]	45.0 [40.0–50.0]	45.0 [40.0–50.0]	0.333
CKI (CrCl < 60 mL/min)	168 (11.3%)	103 (9.9%)	54 (13.3%)	0.060
Preoperative Hb, g/dL	13.3 ± 1.7	13.4 ± 1.7	13.1 ± 1.8	0.004
EuroSCORE II, %	1.44 [0.95–2.25]	1.33 [0.92–2.11]	1.56 [1.07–2.46]	<0.001
**Operative characteristics**				
Aortic CCT, min	63.0 [49.0–80.0]	61.0 [47.0–77.0]	65.5 [52.0–86.0]	<0.001
CPB duration, min	105.0 [84.0–128.0]	101.0 [80.0–123.0]	114.0 [90.0–138.8]	<0.001
Number of grafts	4 [3–4]	3 [3–4]	4 [3–5]	0.004

Data are presented as mean ± standard deviation, median [interquartile range], or n (%). Comparisons between LOS groups include only patients who survived to 30 days (n = 1449); the entire-cohort column additionally includes the 44 patients who died and is descriptive. *p* values compare LOS ≤ 7 days with LOS > 7 days using Welch’s *t*-test, the Mann–Whitney U test, Pearson’s chi-square test, or Fisher’s exact test, as appropriate. BMI, body mass index; CCT, aortic cross-clamp time; CKI, chronic kidney impairment; COPD, chronic obstructive pulmonary disease; CPB, cardiopulmonary bypass; CrCl, creatinine clearance; Hb, hemoglobin; LOS, length of stay; LVEF, left ventricular ejection fraction.

**Table 2:** Postoperative characteristics and outcomes according to LOS category among 30-day survivors.

Variable	LOS ≤ 7 Days *n* = 1043	LOS > 7 Days *n* = 406	*p*	30-Day Deaths *n* = 44	Entire Cohort *n* = 1493
ICU length of stay, days	1.0 [1.0–1.0]	2.0 [1.0–4.0]	<0.001	2.0 [1.0–6.0]	1.0 [1.0–2.0]
POAF	132 (12.7%)	126 (31.0%)	<0.001	20 (45.5%)	278 (18.6%)
Postoperative infection	111 (10.6%)	121 (29.8%)	<0.001	23 (52.3%)	255 (17.1%)
New postoperative dialysis	3 (0.3%)	6 (1.5%)	0.018	22 (50.0%)	31 (2.1%)
Prolonged mechanical ventilation	30 (2.9%)	72 (17.7%)	<0.001	30 (68.2%)	132 (8.8%)
Postoperative vasoactive/inotropic support	173 (16.6%)	146 (36.0%)	<0.001	33 (75.0%)	352 (23.6%)
IABP support	17 (1.6%)	32 (7.9%)	<0.001	27 (61.4%)	76 (5.1%)
ECMO support	1 (0.1%)	2 (0.5%)	0.191	11 (25.0%)	14 (0.9%)
RBC transfusion	854 (81.9%)	325 (80.0%)	0.422	40 (90.9%)	1219 (81.6%)
Cerebrovascular event	9 (0.9%)	13 (3.2%)	0.001	15 (34.1%)	37 (2.5%)

*p* values compare LOS ≤ 7 days with LOS > 7 days among patients who survived to 30 days. The mortality and entire-cohort columns are descriptive. Because postoperative LOS was the grouping variable, it is not repeated in the body of the table. Postoperative LOS was 6.0 [5.0–7.0] days in the LOS ≤ 7 days group and 10.0 [8.0–14.0] days in the LOS > 7 days group. In the 30-day mortality group, the recorded interval from surgery to death was 6.5 [1.8–15.0] days; postoperative LOS in the entire cohort was 7.0 [6.0–8.0] days. ECMO, extracorporeal membrane oxygenation; IABP, intra-aortic balloon pump; ICU, intensive care unit; LOS, length of stay; POAF, postoperative atrial fibrillation; RBC, red blood cell.

**Table 3:** Multivariable models of postoperative hospital LOS among 30-day survivors (n = 1449).

Factor	LOS in Days IRR (95% CI); *p*	LOS > 7 Days aOR (95% CI); *p*
**Panel A. Prespecified primary perioperative model**		
Age, per 10-year increase	1.01 (0.97–1.06); *p* = 0.574	1.15 (1.00–1.33); *p* = 0.053
Female sex	0.98 (0.88–1.09); *p* = 0.754	0.80 (0.57–1.11); *p* = 0.182
BMI, per 5 kg/m^2^ increase	1.05 (1.01–1.10); *p* = 0.014	1.16 (1.01–1.34); *p* = 0.041
Diabetes mellitus	1.07 (1.00–1.14); *p* = 0.036	1.24 (0.97–1.60); *p* = 0.083
Hypertension	1.09 (1.02–1.17); *p* = 0.011	1.26 (0.98–1.62); *p* = 0.075
COPD	1.21 (1.06–1.38); *p* = 0.005	1.53 (1.08–2.16); *p* = 0.016
LVEF, per 5-percentage-point decrease	1.04 (1.01–1.07); *p* = 0.003	1.07 (0.98–1.16); *p* = 0.128
CKI (CrCl < 60 mL/min)	1.09 (0.95–1.24); *p* = 0.230	1.16 (0.78–1.71); *p* = 0.470
Preoperative Hb, per 1 g/dL decrease	1.04 (1.01–1.06); *p* = 0.002	1.09 (1.01–1.17); *p* = 0.018
CPB duration, per 30-min increase	1.11 (1.07–1.15); *p* < 0.001	1.35 (1.22–1.50); *p* < 0.001
**Panel B. Expanded post hoc hospitalization-level sensitivity model (terms of interest)**		
Documented smoking	1.04 (0.96–1.12); *p* = 0.385	1.21 (0.84–1.73); *p* = 0.310
Number of grafts, per additional graft	0.96 (0.93–1.00); *p* = 0.045	0.93 (0.78–1.10); *p* = 0.386
Postoperative infection	1.45 (1.30–1.61); *p* < 0.001	2.54 (1.84–3.51); *p* < 0.001
POAF	1.21 (1.10–1.33); *p* < 0.001	2.38 (1.74–3.25); *p* < 0.001
New postoperative dialysis	1.55 (1.06–2.28); *p* = 0.025	0.73 (0.13–4.15); *p* = 0.722
Prolonged mechanical ventilation	1.52 (1.32–1.75); *p* < 0.001	4.88 (2.98–8.01); *p* < 0.001
Any RBC transfusion (yes/no)	0.89 (0.82–0.98); *p* = 0.012	0.71 (0.51–0.98); *p* = 0.038
Cerebrovascular event	0.84 (0.69–1.02); *p* = 0.084	1.74 (0.67–4.53); *p* = 0.258

Panel A presents the prespecified negative binomial model for LOS in days and the complementary logistic regression model for LOS > 7 days; both models included the 10 factors shown. Panel B presents the terms of interest from an expanded post hoc model containing the same 10 factors and all eight additional terms shown. Both panels included all 1449 survivors with complete data. Reference categories were male sex, absence of each listed comorbidity or postoperative event, and CrCl ≥ 60 mL/min. The >7-day threshold was the survivor-cohort median and was center-specific. Postoperative events were coded as ever/never over the entire index hospitalization, and exact onset times were unavailable. Panel B estimates are adjusted sensitivity estimates representing contemporaneous hospitalization-level associations; they should not be interpreted as temporal, time-specific predictive, or causal effects. In a separate transfusion-dose sensitivity analysis, the IRR per two-unit increase in recorded RBC units was 0.98 (95% CI, 0.95–1.00; *p* = 0.037), and the aOR for LOS > 7 days was 1.00 (95% CI, 0.90–1.11; *p* = 0.938). Because transfusion timing was unavailable, these coefficients should not be interpreted as protective, harmful, temporal, or causal effects. New postoperative dialysis occurred in only nine survivors; the corresponding estimates are imprecise. aOR, adjusted odds ratio; BMI, body mass index; CKI, chronic kidney impairment; COPD, chronic obstructive pulmonary disease; CPB, cardiopulmonary bypass; CrCl, creatinine clearance; Hb, hemoglobin; IRR, incidence rate ratio; LOS, length of stay; LVEF, left ventricular ejection fraction; POAF, postoperative atrial fibrillation; RBC, red blood cell.

**Table 4:** Adjusted associations of preoperative characteristics with POAF and postoperative infection, and exploratory associations with 30-day mortality.

Factor	POAF aOR (95% CI); *p*	Infection aOR (95% CI); *p*	Exploratory 30-Day Mortality aOR (95% CI); *p*
Age, per 10-year increase	1.67 (1.40–1.98); *p* < 0.001	0.94 (0.80–1.11); *p* = 0.500	1.53 (1.05–2.23); *p* = 0.026
Female sex	0.98 (0.69–1.41); *p* = 0.928	1.38 (0.97–1.95); *p* = 0.074	1.48 (0.72–3.04); *p* = 0.289
BMI, per 5 kg/m^2^ increase	0.94 (0.80–1.11); *p* = 0.475	1.29 (1.10–1.52); *p* = 0.002	1.01 (0.71–1.45); *p* = 0.947
Diabetes mellitus	1.11 (0.84–1.46); *p* = 0.469	1.51 (1.12–2.04); *p* = 0.007	1.27 (0.65–2.48); *p* = 0.479
Hypertension	1.50 (1.12–2.00); *p* = 0.007	1.41 (1.04–1.91); *p* = 0.025	1.82 (0.89–3.70); *p* = 0.099
COPD	1.28 (0.87–1.87); *p* = 0.211	1.23 (0.82–1.84); *p* = 0.310	1.84 (0.87–3.88); *p* = 0.110
LVEF, per 5-percentage-point decrease	1.05 (0.95–1.15); *p* = 0.346	1.20 (1.09–1.33); *p* < 0.001	1.35 (1.09–1.68); *p* = 0.006
Preoperative CKI (CrCl < 60 mL/min)	0.81 (0.53–1.25); *p* = 0.344	0.90 (0.56–1.45); *p* = 0.670	1.41 (0.65–3.07); *p* = 0.387
Preoperative Hb, per 1 g/dL decrease	0.98 (0.90–1.06); *p* = 0.561	1.10 (1.01–1.20); *p* = 0.023	1.19 (1.00–1.42); *p* = 0.049
CPB duration, per 30-min increase	—	—	1.77 (1.49–2.11); *p* < 0.001

All three models used the entire cohort (n = 1493), comprising 278 POAF events, 255 postoperative infections, and 44 deaths within 30 days. Reference categories were male sex, absence of diabetes, hypertension, and COPD, and CrCl ≥ 60 mL/min. POAF and infection were analyzed using separate nine-covariate preoperative logistic regression models; perioperative sensitivity models additionally including CPB duration are presented in Supplementary Table S4. The mortality column presents the exploratory 10-parameter Firth model retained for descriptive comparison. With 44 deaths, there were 4.4 events per parameter; individual coefficients should not be interpreted as stable confirmatory estimates, and the model is not a validated prediction model. The parsimonious mortality model and bootstrap internal validation are presented in Supplementary Table S6. aOR, adjusted odds ratio; BMI, body mass index; CKI, chronic kidney impairment; COPD, chronic obstructive pulmonary disease; CPB, cardiopulmonary bypass; CrCl, creatinine clearance; Hb, hemoglobin; LVEF, left ventricular ejection fraction; POAF, postoperative atrial fibrillation.

## Data Availability

The data supporting the findings of this study are available from the corresponding author upon reasonable request. The data are not publicly available due to patient privacy and ethical restrictions.

## Supplementary Materials

The following supporting information can be downloaded at: https://www.mdpi.com/article/10.3390/healthcare14182968/s1, Supplementary Figures S1 and S2 and Supplementary Tables S1–S8 present the alternative and entire-cohort LOS models; model diagnostics and functional-form tests for continuous covariates; the CPB sensitivity and POAF–mortality analyses; detailed infection coding; variable-level data completeness; the sensitivity analysis using categorical year of surgery; and the auxiliary EuroSCORE II and parsimonious mortality analyses. Because the primary model results are presented in Table 3 and Table 4 and Figure 2, Figure 3, Figure 4 and Figure 5, they were not repeated in the Supplementary Materials.
