# Supplementary material for: Factors Associated with Hospital Length of Stay After Coronary Artery Bypass Grafting: Perioperative Characteristics and Postoperative Complication Burden
Source: Healthcare (Basel). 2026 Sep 11;14(18):2968. doi: 10.3390/healthcare14182968 (PMC13606283; doi:10.3390/healthcare14182968)
Supplement: Supplementary file 1 [file healthcare-14-02968-s001.zip › healthcare-4541446-supplementary.pdf]

Factors Associated with Hospital Stay After Coronary Artery Bypass Grafting: Perioperative Characteristics and Postoperative Complication Burden

Supplementary Material

Analytical definitions and focused content

The primary analysis modelled postoperative hospital LOS from operation to discharge from the index admission among 30-day survivors using negative binomial regression; readmission days were not included. LOS >7 days was complementary and centre-specific. Main-text Tables 3–4 and Figures 2–5 report the principal and focused clinical results. This supplement is restricted to alternative distributions, whole-cohort analyses, model diagnostics, perioperative sensitivity analyses, detailed infection coding, data completeness, categorical operation-year sensitivity, and ancillary EuroSCORE II and parsimonious mortality analyses.

The prespecified LOS adjustment set comprised age, sex, BMI, diabetes, hypertension, COPD, LVEF, CrCl <60 mL/min, haemoglobin, and CPB time. It represented demographic characteristics, major comorbidity, physiological reserve, and operative burden and was not selected using univariable P values or stepwise procedures. Cross-clamp and CPB times were not entered together because of collinearity. Infection-site models retained the same covariates and other postoperative events. Mortality analyses were exploratory because only 44 deaths occurred.

Denominator and reference guide. Primary and postoperative-event LOS models used 1449 30-day survivors; full-cohort POAF, infection, mortality, and EuroSCORE II analyses used 1493 patients. Relevant event counts were 278 for POAF, 255 for postoperative infection, and 44 for 30-day mortality. Each table caption or footnote states the exact model sample, event count where applicable, and categorical reference groups.

Supplementary figures

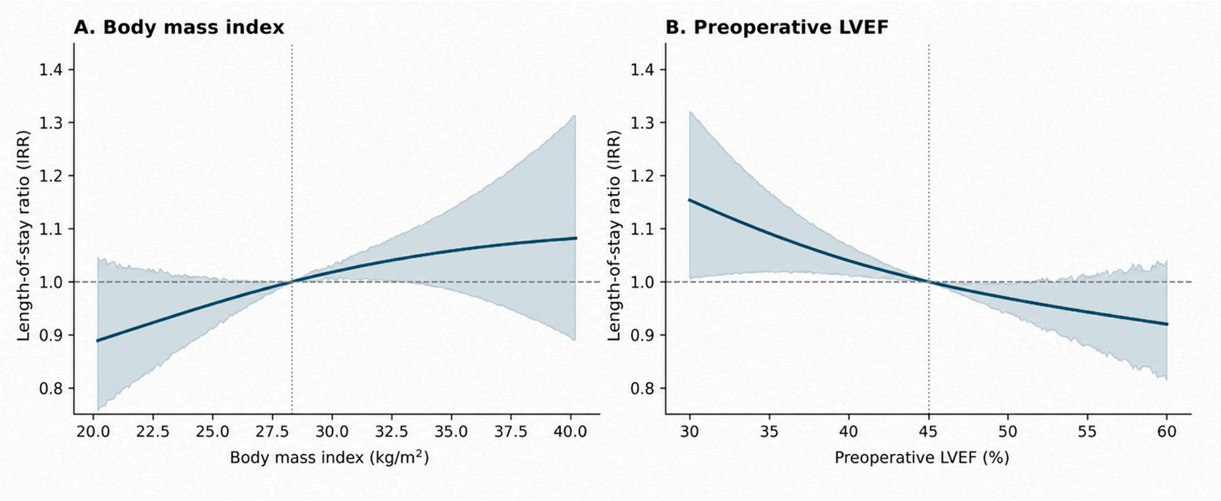

Supplementary Figure S1. Restricted cubic spline models (3 degrees of freedom) for BMI (A) and preoperative LVEF (B) in the negative binomial LOS model among 30-day survivors (*n* = 1449). Curves show incidence rate ratios with 95% confidence intervals. Functional-form comparisons for age, BMI, LVEF, haemoglobin, and CPB time are reported together in Supplementary Table S3; neither BMI nor LVEF showed evidence of non-linearity. Both complete-case models used all 1449 survivors and the prespecified adjustment set. Categorical reference groups were male sex and absence of diabetes, hypertension, COPD, and renal dysfunction.

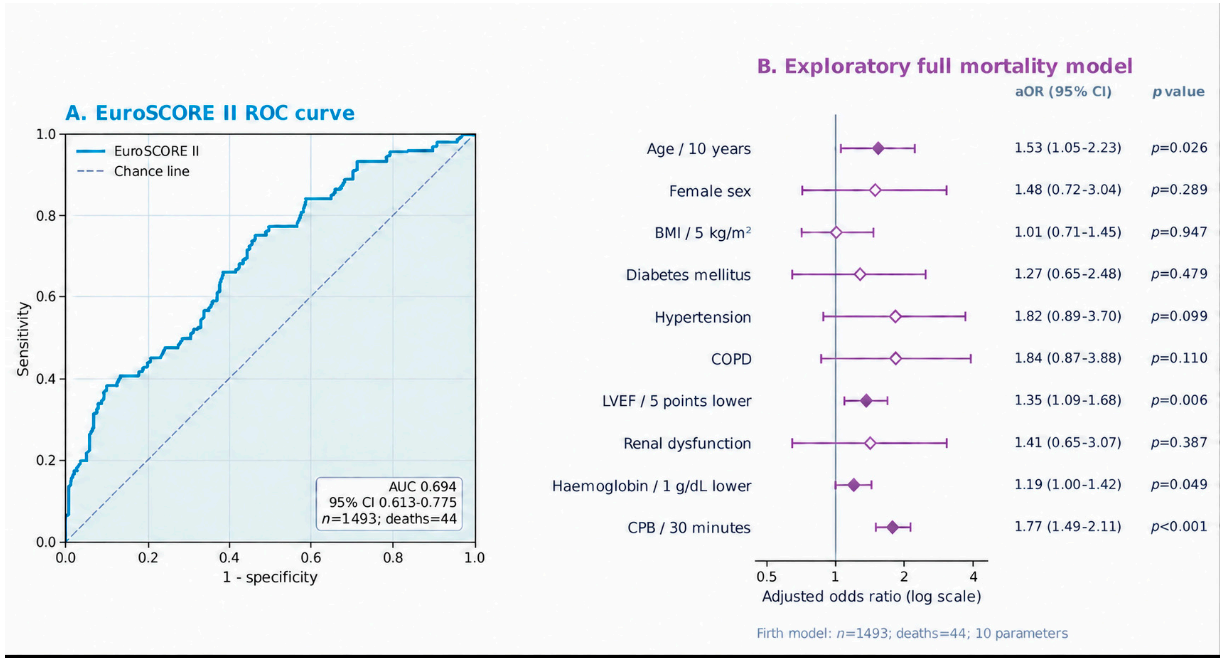

Supplementary Figure S2. Ancillary EuroSCORE II discrimination and exploratory associations with 30-day mortality. (A) Receiver operating characteristic curve for EuroSCORE II in the full cohort (n = 1493; 44 deaths); AUC 0.694 (95% CI 0.613–0.775; DeLong method). The dashed diagonal represents chance discrimination. (B) Adjusted odds ratios from the exploratory 10-parameter Firth logistic model using all 1493 complete cases (44 deaths; 4.4 events per parameter). Filled diamonds indicate $p < 0.05$, open diamonds indicate $p \geq 0.05$, and the vertical line is the null (aOR = 1.0). Panel B is descriptive and should not be interpreted as a stable, confirmatory, or validated prediction model; the parsimonious mortality model and bootstrap internal validation are reported in Supplementary Table S6.

Supplementary Table S1. Alternative continuous-LOS models among 30-day survivors (n = 1449); principal negative-binomial and LOS >7-day estimates are reported in main-text Table 3 and Figure 2.

Factor	Log-linear GMR (95% CI)	*p*	Median change days (95% CI)	*p*	75th-percentile change days (95% CI)	*p*
Age, per 10-year increase	1.02 (1.00–1.05)	0.117	0.16 (0.05–0.27)	0.004	0.18 (−0.02–0.38)	0.078
Female sex	0.97 (0.92–1.03)	0.323	−0.11 (−0.37–0.15)	0.395	−0.26 (−0.70–0.18)	0.251
Body mass index, per 5-kg/m² increase	1.03 (1.01–1.06)	0.018	0.16 (0.05–0.28)	0.004	0.13 (−0.07–0.32)	0.210
Diabetes mellitus	1.05 (1.01–1.09)	0.025	0.07 (−0.12–0.26)	0.490	0.24 (−0.09–0.58)	0.154
Hypertension	1.05 (1.01–1.10)	0.015	0.12 (−0.07–0.32)	0.223	0.29 (−0.05–0.64)	0.093
Chronic obstructive pulmonary disease	1.12 (1.03–1.21)	0.008	0.21 (−0.08–0.50)	0.156	0.96 (0.45–1.47)	<0.001
LVEF, per 5-percentage-point decrease	1.02 (1.01–1.04)	0.002	0.07 (0.00–0.13)	0.043	0.15 (0.03–0.27)	0.012
Preoperative renal dysfunction (CrCl <60 mL/min)	1.06 (0.98–1.14)	0.182	0.03 (−0.29–0.36)	0.832	0.44 (−0.12–1.01)	0.126
Preoperative haemoglobin, per 1-g/dL decrease	1.02 (1.01–1.04)	0.002	0.07 (0.02–0.13)	0.012	0.11 (0.01–0.21)	0.025
CPB time, per 30-minute increase	1.07 (1.05–1.10)	<0.001	0.29 (0.21–0.37)	<0.001	0.62 (0.47–0.77)	<0.001

GMR, geometric mean ratio. Quantile-regression coefficients are conditional changes in days. All models used the same 10 prespecified covariates and 1449 complete cases. The principal negative-binomial and complementary LOS >7-day models are not repeated here because their complete estimates appear in main-text Table 3 and Figure 2.

Supplementary Table S2. Whole-cohort sensitivity models (*n* = 1493), including recorded time to death for 44 in-hospital deaths.

Factor	Negative binomial IRR (95% CI)	*P*	Log-linear GMR (95% CI)	*P*	Median change days (95% CI)	*P*	75th-percentile change days (95% CI)	*P*
Age, per 10-year increase	1.01 (0.96–1.06)	0.645	1.01 (0.98–1.04)	0.417	0.17 (0.06–0.28)	0.002	0.22 (0.02–0.43)	0.031
Female sex	0.99 (0.89–1.11)	0.920	0.98 (0.91–1.05)	0.560	−0.09 (−0.35–0.17)	0.494	−0.07 (−0.53–0.38)	0.748
Body mass index, per 5-kg/m² increase	1.05 (1.00–1.09)	0.038	1.02 (0.99–1.05)	0.113	0.16 (0.04–0.27)	0.007	0.07 (−0.14–0.27)	0.509
Diabetes mellitus	1.09 (1.02–1.16)	0.010	1.07 (1.03–1.13)	0.002	0.12 (−0.07–0.32)	0.213	0.39 (0.05–0.74)	0.025
Hypertension	1.10 (1.03–1.18)	0.006	1.05 (1.01–1.10)	0.024	0.12 (−0.08–0.32)	0.242	0.33 (−0.02–0.69)	0.063
Chronic obstructive pulmonary disease	1.22 (1.07–1.39)	0.003	1.12 (1.02–1.23)	0.014	0.19 (−0.10–0.48)	0.204	1.26 (0.74–1.78)	<0.001
LVEF, per 5-percentage-point decrease	1.04 (1.01–1.06)	0.005	1.02 (1.00–1.03)	0.036	0.05 (−0.01–0.12)	0.116	0.16 (0.04–0.28)	0.010
Preoperative renal dysfunction (CrCl <60 mL/min)	1.07 (0.93–1.24)	0.312	1.05 (0.96–1.15)	0.295	−0.04 (−0.36–0.28)	0.791	0.39 (−0.19–0.96)	0.192
Preoperative haemoglobin, per 1-g/dL decrease	1.03 (1.00–1.05)	0.020	1.01 (0.99–1.03)	0.204	0.07 (0.01–0.13)	0.019	0.08 (−0.02–0.18)	0.115
CPB time, per 30-minute increase	1.09 (1.05–1.13)	<0.001	1.04 (1.00–1.07)	0.031	0.23 (0.15–0.31)	<0.001	0.59 (0.45–0.74)	<0.001

For patients who died, the recorded interval from surgery to death was used without modification; death was not automatically classified as LOS >7 days. The whole-cohort negative binomial model estimated alpha = 0.154. All models used 1493 complete cases; reference categories were male sex and absence of diabetes, hypertension, COPD, and renal dysfunction.

Supplementary Table S3. Distributional choice, sensitivity, linearity, and collinearity diagnostics for the primary LOS models among 30-day survivors (*n* = 1449).

Test	Result	Interpretation
Overdispersion parameter (alpha), negative binomial	0.136	Substantial overdispersion
Pearson chi-square / degrees of freedom in the Poisson model	3.60	Poisson assumption violated
Likelihood-ratio test of negative binomial versus Poisson	LR=1,083.5; df=1; $p<0.001$	Strong support for negative binomial modelling
Zero-truncated negative binomial sensitivity	alpha=0.142; all core IRRs within 0.01	Near-identical effect sizes
Age: 3-df spline versus linear term	Count LR=0.95, df=2, p=0.621; >7-day LR=2.72, df=2, p=0.257	No evidence of non-linearity
Body mass index: 3-df spline versus linear term	Count LR=2.51, df=2, p=0.285; >7-day LR=0.45, df=2, p=0.798	No evidence of non-linearity
Left ventricular ejection fraction: 3-df spline versus linear term	Count LR=1.04, df=2, p=0.596; >7-day LR=2.48, df=2, p=0.290	No evidence of non-linearity
Preoperative haemoglobin: 3-df spline versus linear term	Count LR=9.10, df=2, p=0.011; >7-day LR=0.98, df=2, p=0.613	Base count-model signal; attenuated after categorical-year adjustment (p=0.114)
Cardiopulmonary bypass time: 3-df spline versus linear term	Count LR=5.19, df=2, p=0.075; >7-day LR=0.23, df=2, p=0.892	No evidence of non-linearity
Joint non-linearity test: all five continuous covariates	Count LR=17.96, df=10, p=0.056; >7-day LR=6.77, df=10, p=0.747	No clear joint improvement over linear terms
Correlation between cross-clamp and CPB times	Pearson r=0.876; Spearman ρ=0.891; $p<0.001$	Strong association
Variance inflation factor with both durations	CPB 4.40; cross-clamp 4.38	Only CPB retained because of collinearity

Each continuous covariate was represented separately by a restricted cubic spline with 3 degrees of freedom and compared with its linear term using a 2-df likelihood-ratio test. The haemoglobin non-linearity signal in the base count model was not retained after categorical-year adjustment. Linear terms were therefore retained for parsimony; their ratios summarize average associations across the observed ranges. Unless otherwise stated, diagnostic models used all 1449 complete-case survivors and the prespecified 10-covariate adjustment set; categorical reference groups were male sex and absence of diabetes, hypertension, COPD, and renal dysfunction.

Supplementary Table S4. Perioperative sensitivity analyses for POAF/infection and sequential POAF-mortality analyses.

Panel A. Perioperative sensitivity models after addition of CPB time

**Factor**	**POAF + CPB aOR (95% CI)**	***p***	**Infection + CPB aOR (95% CI)**	***p***
Age, per 10-year increase	1.65 (1.39–1.96)	<0.001	0.94 (0.80–1.11)	0.443
Female sex	1.00 (0.70–1.44)	0.988	1.41 (0.99–2.00)	0.057
Body mass index, per 5-kg/m^2 increase	0.94 (0.80–1.11)	0.457	1.29 (1.10–1.52)	0.002
Diabetes mellitus	1.09 (0.83–1.44)	0.540	1.49 (1.10–2.01)	0.009
Hypertension	1.47 (1.10–1.97)	0.010	1.39 (1.03–1.88)	0.034
Chronic obstructive pulmonary disease	1.27 (0.87–1.86)	0.220	1.23 (0.82–1.85)	0.309
LVEF, per 5-percentage-point decrease	1.04 (0.95–1.14)	0.413	1.19 (1.08–1.32)	<0.001
Preoperative renal dysfunction (CrCl <60 mL/min)	0.82 (0.53–1.25)	0.353	0.89 (0.56–1.43)	0.635
Preoperative haemoglobin, per 1-g/dL decrease	0.98 (0.90–1.06)	0.566	1.10 (1.01–1.20)	0.023
CPB time, per 30-minute increase	1.14 (1.03–1.27)	0.012	1.13 (1.01–1.26)	0.026

Each column is a separate complete-case multivariable logistic model using the full cohort (n = 1493): 278 POAF events and 255 postoperative infections. Reference categories were male sex, no diabetes, no hypertension, no COPD, and CrCl >=60 mL/min. The corresponding preoperative models are reported in main-text Table 4 and Figure 3. CPB, cardiopulmonary bypass; POAF, postoperative atrial fibrillation; aOR, adjusted odds ratio.

Supplementary Table S4 (continued). Focused POAF and postoperative-infection analyses.

Panel B. Sequential association between POAF and 30-day mortality

Model	Effect for POAF (95% CI)	*p*	Variables in the model
Unadjusted risk ratio (Katz)	RR 3.64 (2.04–6.50)	<0.001	— (2 × 2 table)
Unadjusted Firth odds ratio	OR 3.86 (2.11–7.05)	<0.001	POAF
Parsimonious Firth model	aOR 2.97 (1.57–5.62)	<0.001	POAF + age + LVEF + renal dysfunction + haemoglobin + CPB time
Full postoperative Firth model	aOR 1.76 (0.78–3.99)	0.172	Parsimonious model + infection + new dialysis + prolonged ventilation

All analyses used the full cohort (n=1493; 44 deaths), and the reference category was no POAF. The risk ratio is the observed mortality-risk ratio; odds ratios were estimated using Firth penalised likelihood. Mortality was 20/278 (7.19%) with POAF and 24/1215 (1.98%) without POAF. Attenuation in the postoperative model indicates overlap and does not establish mediation or causality.

Rare-event sensitivity for the admission-level LOS >7-day models shown in main-text Figure 4: Firth aORs were 2.49 (95% CI 1.81–3.44; p<0.001) for infection, 2.39 (1.76–3.25; p<0.001) for POAF, 0.64 (0.12–3.52; p=0.611) for new dialysis, and 4.84 (2.96–7.93; p<0.001) for prolonged mechanical ventilation. These estimates are retained as a sensitivity note rather than a duplicate table.

Supplementary Table S5. Recorded anatomical patterns among the 255 patients with postoperative infection.

Code	Recorded anatomical pattern	All cohort, *n* (% of infections)	30-day survivors, *n*
1	Superficial sternal wound infection	5 (2.0%)	5
2	Saphenous harvest-site infection	68 (26.7%)	60
3	Pneumonia	56 (22.0%)	51
4	Mediastinitis (with concurrent sternal involvement)	15 (5.9%)	14
5	Mediastinitis + saphenous harvest-site infection	47 (18.4%)	47
6	Mediastinitis + pneumonia	27 (10.6%)	22
7	Superficial sternal wound infection + pneumonia	37 (14.5%)	33

All infection records had a site code. Sepsis was assigned to the documented primary focus and was not a separate site. Mediastinitis and its concurrent sternal involvement were not treated as independent events. Percentages in the all-cohort column use the 255 infected patients as the denominator; the survivor column reports counts within the 1449-patient survivor cohort.

Supplementary Table S6. Ancillary EuroSCORE II performance and exploratory mortality modelling. Sections A–D use the full cohort (n=1493; 44 deaths). Section E reports outcome-specific denominators: postoperative infection, n=1493/255 events; LOS >7 days, n=1449/406 events; postoperative stroke, n=1493/37 events.

**Analysis / metric**	**Estimate**	**95% CI**	***p* / interpretation**
**A. Fixed EuroSCORE II: local apparent performance**			
Observed 30-day mortality	44/1493 (2.95%)	-	All deaths during index admission
Expected deaths / mean predicted risk	30.57 / 2.05%	-	Sum / mean EuroSCORE II prediction
Observed-to-expected ratio	1.44	1.05-1.93	Underprediction in this cohort
EuroSCORE II AUC	0.694	0.613-0.775	Moderate apparent discrimination
Age AUC	0.645	0.551-0.731	Exploratory single-marker comparison
Preoperative LVEF AUC	0.628	0.539-0.714	Exploratory single-marker comparison
Brier score	0.0278	0.0199-0.0359	Bootstrap 95% CI
Scaled Brier improvement	2.72%	-	Limited absolute improvement
Calibration intercept	0.385	0.080-0.691	Slope fixed at 1
Calibration slope	1.008	0.663-1.352	Local apparent calibration estimate; interpret cautiously
**B. Parsimonious Firth mortality model**			
EuroSCORE II, per doubling	OR 2.01	1.55-2.62	p<0.001
CPB time, per 30 minutes	OR 1.78	1.49-2.14	p<0.001
**C. Continuous-operation-year sensitivity**			
EuroSCORE II, per doubling	OR 1.87	1.43-2.44	p<0.001
CPB time, per 30 minutes	OR 1.74	1.45-2.09	p<0.001
Operation year, per later year	OR 0.78	0.63-0.95	p=0.015
**D. Bootstrap internal validation (apparent -> corrected)**			
AUC	0.801 -> 0.799	0.737-0.868	Mean optimism 0.003

Analysis / metric	Estimate	95% CI	*p* / interpretation
Brier score	0.0262 -> 0.0268	0.0195-0.0338	Mean optimism 0.0006
Calibration intercept	-0.025 -> -0.008	-0.309-0.341	Mean optimism -0.017
Calibration slope	1.006 -> 0.992	0.777-1.268	Mean optimism 0.014
**Analysis / metric**	**Estimate**	**95% CI**	***p* / interpretation**
**E. Post hoc EuroSCORE II associations**			
Postoperative infection (*n*=1493; 255 events)	OR 1.40; AUC 0.580	1.22-1.61	*p*<0.001
LOS >7 days (*n*=1449; 406 events)	OR 1.39; AUC 0.569	1.22-1.58	*p*<0.001
Postoperative stroke (*n*=1493; 37 events)	OR 1.76; AUC 0.654	1.33-2.34	*p*<0.001; AUC CI 0.560-0.747

Sections A and E describe the fixed external EuroSCORE II in this single-centre cohort; they are ancillary and do not constitute external validation. Sections B–D describe the two-parameter Firth model (log2 EuroSCORE II and CPB time), its continuous-year sensitivity, and 2000-sample bootstrap internal validation. The exploratory 10-parameter model remains in Supplementary Figure S2B because 44 deaths yield only 4.4 events per parameter. Section E uses the outcome-specific denominators and event counts stated in the caption. None of these analyses establishes external validity or supports use of the score as a stand-alone classifier for non-mortality outcomes.

Supplementary Table S7. Variable-level data completeness in the included cohort.

Variable	Source field	Available n	Missing n (%)
Postoperative infection	infect	1493	0 (0.0%)
Operation year	operation_date	1493	0 (0.0%)
Infection localisation	Localization_of_infection	1493	0 (0.0%)
Postoperative hospital days during the index admission	hos_stay	1493	0 (0.0%)
Sex	sex	1493	0 (0.0%)
Weight	weight	1493	0 (0.0%)
Intra-aortic balloon pump use	iabp	1493	0 (0.0%)
Height	height	1493	0 (0.0%)
Body mass index	bmi	1493	0 (0.0%)
Hypertension	ht	1493	0 (0.0%)
Preoperative serum creatinine	precrea	1493	0 (0.0%)
Creatinine clearance	crcl	1493	0 (0.0%)
Preoperative haemoglobin	prehb	1493	0 (0.0%)
Cardiopulmonary bypass time	cpbtime	1493	0 (0.0%)
New postoperative dialysis	newdial	1493	0 (0.0%)
Postoperative atrial fibrillation	postaf	1493	0 (0.0%)
Prolonged mechanical ventilation	prolint	1493	0 (0.0%)
Intensive-care-unit stay	icu_stay	1493	0 (0.0%)
Vasoactive/inotropic support	inotrope	1493	0 (0.0%)
Extracorporeal membrane oxygenation	ecmo	1493	0 (0.0%)
Chronic obstructive pulmonary disease	copd	1493	0 (0.0%)
Diabetes mellitus	dm	1493	0 (0.0%)
EuroSCORE II	Euro_II	1493	0 (0.0%)
Aortic cross-clamp time	cctime	1493	0 (0.0%)
Left ventricular ejection fraction	LVEF	1493	0 (0.0%)
Thirty-day mortality	mort30d	1493	0 (0.0%)
Postoperative stroke	stroke	1493	0 (0.0%)
Smoking status	smoking	1493	0 (0.0%)
Graft number	Graft_number	1493	0 (0.0%)

Variable	Source field	Available n	Missing n (%)
Age	age	1493	0 (0.0%)
Recorded erythrocyte suspension units	Blood_transfusion	1493	0 (0.0%)
Renal dysfunction (CrCl <60 mL/min; derived)	crcl	1493	0 (0.0%)
LOS >7 days (derived among 30-day survivors)	hos_stay + mort30d	1449	0 (0.0%)

All 31 source variables in the analysis dataset were complete, as were the derived renal-dysfunction and LOS variables. LOS >7 days was defined only among the 1449 30-day survivors; its smaller denominator reflects the survivor-specific outcome definition and not missing data. No imputation was performed.

Supplementary Table S8. Categorical operation-year sensitivity analyses among 30-day survivors (n = 1449).

Panel A. Annual distribution of postoperative hospital length of stay

Operation year	*n*	LOS, median [IQR], days	LOS >7 days, *n* (%)
2020	174	7 [6–14]	85 (48.9%)
2021	246	7 [6–10]	113 (45.9%)
2022	239	7 [6–8]	114 (47.7%)
2023	258	6 [6–7]	58 (22.5%)
2024	350	7 [5–7]	21 (6.0%)
2025	182	6 [5–7]	15 (8.2%)

Panel B. Categorical-year-adjusted multivariable models

Factor	Adjusted IRR (95% CI)	*p*	Adjusted aOR for LOS >7 days (95% CI)	*p*
Age, per 10 years	1.01 (0.97–1.06)	0.599	1.17 (1.00–1.36)	0.052
Female sex	1.00 (0.90–1.11)	0.959	0.77 (0.54–1.11)	0.158
BMI, per 5 kg/m²	1.05 (1.01–1.10)	0.010	1.23 (1.05–1.44)	0.011
Diabetes mellitus	1.06 (0.99–1.14)	0.093	0.94 (0.71–1.25)	0.673
Hypertension	1.09 (1.02–1.16)	0.007	1.32 (1.00–1.75)	0.048
COPD	1.16 (1.02–1.33)	0.028	1.43 (0.98–2.10)	0.064
LVEF, per 5 percentage points lower	1.03 (1.01–1.06)	0.003	1.07 (0.98–1.17)	0.140
Renal dysfunction	1.05 (0.92–1.21)	0.476	1.04 (0.67–1.62)	0.850
Haemoglobin, per 1 g/dL lower	1.03 (1.01–1.05)	0.007	1.04 (0.96–1.12)	0.384
CPB time, per 30 minutes	1.09 (1.06–1.13)	<0.001	1.38 (1.23–1.54)	<0.001
Operation year 2021 vs 2020	0.70 (0.61–0.80)	<0.001	0.99 (0.66–1.48)	0.944
Operation year 2022 vs 2020	0.77 (0.66–0.90)	<0.001	1.11 (0.73–1.67)	0.625
Operation year 2023 vs 2020	0.65 (0.57–0.74)	<0.001	0.37 (0.24–0.57)	<0.001
Operation year 2024 vs 2020	0.58 (0.51–0.67)	<0.001	0.07 (0.04–0.12)	<0.001
Operation year 2025 vs 2020	0.71 (0.59–0.86)	<0.001	0.11 (0.06–0.21)	<0.001

All estimates used 1449 complete cases. Operation year was entered as a six-level categorical variable (2020 reference). Global likelihood-ratio tests were LR=158.50, df=5, $p<0.001$ for the count model and LR=250.76, df=5, $p<0.001$ for LOS >7 days. The maximum absolute change in a core estimate was 3.7% for the count model and 24.5% for the binary model. IRR, incidence rate ratio; aOR, adjusted odds ratio; LVEF, left ventricular ejection fraction; CPB, cardiopulmonary bypass. Reference categories for other binary covariates were male sex and absence of diabetes, hypertension, COPD, and renal dysfunction.
